# Available prediction scores of conversion for laparoscopic rectal cancer surgery seem to be unsuitable for nowadays rectal cancer management

**DOI:** 10.1186/s12893-022-01617-9

**Published:** 2022-05-10

**Authors:** Hamza Sekkat, Amine Souadka, Lise Courtot, Ali Rafik, Laila Amrani, Amine Benkabbou, Pierre Peyrafort, Urs Giger-Pabst, Elias Karam, Raouf Mohsine, Anass M. Majbar, Mehdi Ouaissi

**Affiliations:** 1 Digestive Surgical Oncology Department, National Institute of Oncology, Ibn Sina University Hospital, Rabat, Morocco; 2 Equipe de Recherche en Oncologie Translationnelle (EROT), Faculty of Medicine and Pharmacy, Mohammed V University in Rabat, Rabat, Morocco; 3 Department of Digestive, Oncological, Endocrine, Hepato-Biliary, Pancreatic and Liver Transplant Surgery, Trousseau Hospital, Chambray les Tours, France; 4 EA4245 Transplantation, Immunologie, Inflammation, Université de Tours, Tours, France

**Keywords:** Rectal neoplasms, Laparoscopy, Conversion, Predictive models

## Abstract

**Introduction:**

This study aimed to externally evaluate the accuracy of four predictive scores for conversion to open surgery after rectal laparoscopic resection. None of the four scores achieved external validation previously.

**Methods:**

This was a retrospective analysis of two prospectively maintained databases from two academic centers in France and Morocco. All consecutive patients who underwent laparoscopic resection for rectal adenocarcinoma between 2005 and 2020 were included. Logistic regression was used to assess the association between the factors present in the four scores and conversion. The accuracy of each score was assessed using the area under the curve (AUC). Observed and predicted conversion rates were compared for each score using the Chi-square goodness-of-fit test.

**Results:**

Four hundred patients were included. There were 264 men (66%) with a mean age of 65.95 years (standard deviation 12.2). The median tumor height was 7 cm (quartiles 4–11) and 29% of patients had low rectal tumors. Conversion rate was 21.75%. The accuracy to predict conversion was low with an AUC lower than 0,62 for the four models. The observed conversion rates were significantly different from the predicted rates, except for one score.

**Conclusions:**

The four models had low accuracy in predicting the conversion to open surgery for laparoscopic rectal resection. There is a need for new well-designed studies, analyzing more specific variables, in a multicentric design to ensure generalizability of the results for daily surgical practice.

## Introduction

The worldwide adoption of laparoscopy as the “Gold Standard” for rectal cancer surgery has been slower than other oncologic digestive procedures such as colonic cancer [1], due to the specific technical difficulties of rectal surgery and the long duration to complete the learning curve [2]. In the first randomized controlled trial comparing laparoscopic and open surgery for colorectal cancer, the conversion rate in the laparoscopic group was 34% [2]. Since then, conversion rates in the literature have decreased but remain relatively high. For example, in the recent COLOR 2 trial, conversion rate was 17% [3].

Conversion to open is used as an indicator for rectal laparoscopic surgery success and to assess the learning curve for individual surgeons [4]. Laparoscopic surgery for rectal cancer is associated with better postoperative outcomes such as less pain, quick recovery, and shorter hospital stay [1]. However, conversion has been associated with worse postoperative outcomes [5]. Therefore, being able to predict the risk of conversion before surgery is important to decide whether laparoscopy is suitable or not and to provide patients with better information about potential surgical outcomes and prognosis.

In the literature, few scores have been proposed to predict the risk of conversion of laparoscopic colorectal surgeries, with only one focusing specifically on rectal cancers [6–9]. The university of Toronto model was developed in 2000 by Schlachta et al. Logistic regression analysis allowed the score creation based on 3 criteria that were found to be strongly associated with conversion: malignancy, patient weight, and surgeon experience [9]. The Cleveland model developed by Tekkis et al. proposed a score including 6 factors linked the most with conversion: body mass index (BMI) American Society of Anesthesiologists (ASA) grade, type of surgery (right-sided, left-sided, low rectal, and others), intra-abdominal fistula, intra-abdominal abscess, and surgeon seniority [8]. Both models were validated within their respective institutions by using a split-sample validation. The Vaccaro model from Argentina was also developed using logistic regression, and included Male gender, Body surface area ≥ 1.8, and rectal disease. Internal validation was fulfilled by using bootstrapping [6]. Zhang et al. from China performed a multiple regression analysis to propose a score made of six variables that were linked to a higher risk of conversion: male gender, surgical experience, abdominal surgery antecedent, BMI, tumor diameter, and tumor invasion or metastasis. The model validation was performed on 50 patients who underwent rectal laparoscopic surgery [7].

Although all these scores were validated internally, external validation remains the first step before applying them broadly in general clinical practice. Therefore, this study aimed to evaluate externally and compare the performance of these four scores, to identify the best conversion predictive score for rectal cancer laparoscopic procedures.

## Methods

This study is reported according to the STROBE (Strengthening the Reporting of Observational Studies in Epidemiology) guidelines for observational studies [10]. This study was approved by the local comity of informatics and liberty (CIL) (n° 2020-067) in Tours, according to French law (Code de la santé publique, Article R1121-1 Modified by Décret n°2017-884 du 9 Mai 2017—art.2) and by the institutional review board of National Institute of Oncology waiving ethical approval according to the Moroccan law Moroccan law (Law 28/13—art.2). All procedures in studies involving human participants were performed according to the ethical standards of the institutional and/or national research committee and with the 1964 Helsinki declaration and its later amendments or comparable ethical standards. Written informed consent was obtained from all individual participants included in the study.

### Study design and setting study

This was a retrospective study conducted from 2 prospectively maintained institutional rectal cancer surgery databases at two academic centers in France (Department of Digestive, Oncological, Endocrine, Hepato-Biliary, Pancreatic, and Liver Transplant Surgery, Trousseau Hospital, Tours) and Morocco (Digestive surgical oncology department at the National Institute of Rabat).

### Participants

The study included all consecutive adult patients (> 18 years-old) who underwent laparoscopic surgery for primary rectal adenocarcinoma (within 15 cm from the anus at rigid endoscopy) with a curative intention between January 2005 and December 2019 in both centers. Patients who underwent a palliative resection, who had a single port technique, or a hand assisted technique or robotic surgery were not included. All patients were treated according to the French guidelines of rectal cancer management [11]. Patients with high rectal adenocarcinoma underwent partial mesorectal excision, with at least 5 cm distal margin. Patients with mid and low rectal adenocarcinomas underwent complete mesorectal excision. When a 1 cm distal margin could be achieved, a sphincter preserving surgery was done. Radiochemotherapy was indicated for T3, T4 or/and node positive mid and low rectal tumors.

### Collection data procedure

All socio-demographic and therapeutic data were extracted for rectal cancer patients: age, gender, tumor location, histological subtype, Tumor-Node-Metastasis (TNM) staging, and treatment. All predictive risk conversion scores and calculating methods were taken from previously published studies [6–8, 9]. Our database included all the information needed to calculate the 4 scores.

The university of Toronto model contains 3 factors: malignancy (0 or 1 point), patient weight (0 to 2 points) and surgeon experience (0 or 1 point) [9]. The total score ranges from 0 to 4 points and is associated with a predictive risk of conversion ranging from 1.1% to 49,7%. The Cleveland model includes 6 factors: body mass index (0 to 0.8 points) American Society of Anesthesiologists grade (0 to 1.8 points), type of surgery (right-sided, left-sided, low rectal, and others) (0 to 2.2 points), intra-abdominal fistula (0 to 1.6 points), intra-abdominal abscess (0 to 1.3 points), and surgeon seniority (0 to 0.4 points) [8]. The total score ranges from 0 to 8 points and is associated with a predictive risk of conversion ranging from 0.2% to 88.1%. The Vaccaro model contains 3 factors: male gender (1 point), Body surface area ≥ 1.8 (1 point), and rectal disease (1 point) [6]. The total score ranges from 0 to 3 points and is associated with a predictive risk of conversion ranging from 3.7% to 24.4%. Finally, Zhang et al. model includes six variables: male gender (6 points), surgical experience (4 points), history of abdominal surgery (5 points), obesity (10 points), tumor diameter (15 points), and tumor invasion or metastasis (21 points). The final score ranges from 0 to 61 points). A score < 14.5 has a predictive risk of conversion of 3.88% while a score > 14.5 has 47.83% risk [7].

### Definition of conversion to open surgery

There was a variation in conversion definitions between the included studies. Tekkis et al. defined conversion as “the need for a midline laparotomy greater than 10 cm, for either completion of the operative procedure for extraction of the specimen'' [8], while Schlachta et al. considered that “Any case that cannot be completed laparoscopically as planned is considered a conversion to open surgery” [9]. The definition in Vaccaro’s study was vague and Zhang et al. did not report a definition in their paper [6, 7]. For this study, conversion to open surgery was defined as any abdominal incision performed for inability to complete the surgical steps by laparoscopy. Enlarging the extraction site incision to remove a bulky tumor was not considered a conversion to an open procedure [12]. This definition is more precise and widely used in the literature [12, 13].

### Statistical analysis

Descriptive analysis was performed for sociodemographic and clinical features. Quantitative variables were expressed as means (with standard deviation) or median (and quartiles) as appropriate. Qualitative data were expressed as numbers and percentages.

The accuracy of the scores refers to the ability to predict conversion to laparotomy was evaluated using the Receiver Operating Characteristics (ROC). The Area Under the Curve (AUC) was calculated for each score to assess the ability to differentiate patients who had conversion to open surgery from those who had no conversion. A score was considered reliable if the AUC was above 0.8 [14]. Predicted conversion rates for each score were calculated using the published scoring system and they were compared to the observed conversion rate in this study using the X2 goodness of fit test [13].

In addition, risk factors for conversion to open surgery were analyzed to assess if the variables included in the four scores were associated with conversion in this series. For univariate analysis, comparisons of continuous variables for two independent samples employed the Student test or Wilcoxon rank-sum test for continuous variables as appropriate, and Chi-square or Fisher exact tests for categorical variables, as appropriate. Variables with p < 0.1 were included in the final multivariate model. Adjusted odds ratios with 95% confidence intervals (CIs) for each variable were calculated as an estimate of the likelihood of having conversion to laparotomy. A p-value of less than 0.05 was considered significant. All the above analyses were performed using the SPSS software version 25.

## Results

### Patients’ characteristics

During the study period, 516 patients had surgical resection for rectal adenocarcinoma in both centers. Among them, 400 patients underwent curative rectal laparoscopic surgery. Eighty-seven patients (21.75%) had conversion to open surgery.

Patients’ characteristics are represented in Table 1. There were 264 men (66%) with a mean age of 65.95 years (standard deviation 12.2). Twenty-seven patients (24.25%) had a BMI above 28 kg/m^2^ and twenty patients (5%) had previous abdominal surgery. The median tumor height was 7 cm (quartiles 4–11) and 29% of patients had low rectal tumors (tumor height ≤ 5 cm). Neoadjuvant radiochemotherapy (50.4 Grey + Capecitabine) was administered to 283 patients (70.75%). Twenty-two patients had T4 stage on the surgical specimen and 25 patients (6.25%) had positive circumferential resection margins. In total, 30 surgeons performed laparoscopic rectal resections. Among 400 laparoscopic rectal resections, 320 were performed by senior surgeons (80%).

**Table 1:** Patients’ characteristics

Age (SD) years	65.95 (12.2)
Sex	
Male	264(66%)
Female	136(34%)
PS	
0–1	387(96.75%)
> 1	13(3.25%)
ASA score	
1–2	343(85.75%)
> 2	50(12.5%)
Missing	7(1.75%)
BMI (Kg/m^2^)	
< 18	14(4.5%)
18—28	283(70.75%)
> 28	97(24.25%)
Missing	6
History of surgery	
Yes	20(5%)
No	380(95%)
Tumor location	
Median (quartiles) cm	7(4–11)
Low	116(29%)
Medium	124(31%)
Hight	155(38.75%)
Missing	5
Neoadjuvant CRT	283(70.75%)
pT Stage	
0–3	377(94.25%)
4	22(5.5%)
Missing	1
M stage	
M0	347(86.75%)
M1	52(13%)
CRM +	25(6.25%)
DRM +	25(6.25%)
Conversion rate	21.75%

*PS* performance status, *ASA* American Score of Anesthesiologists, *CRT* chemoradiotherapy, *CRM* circumferential resection margin, *DRM* distal resection margin

### Scores performances

**Fig. 1:**
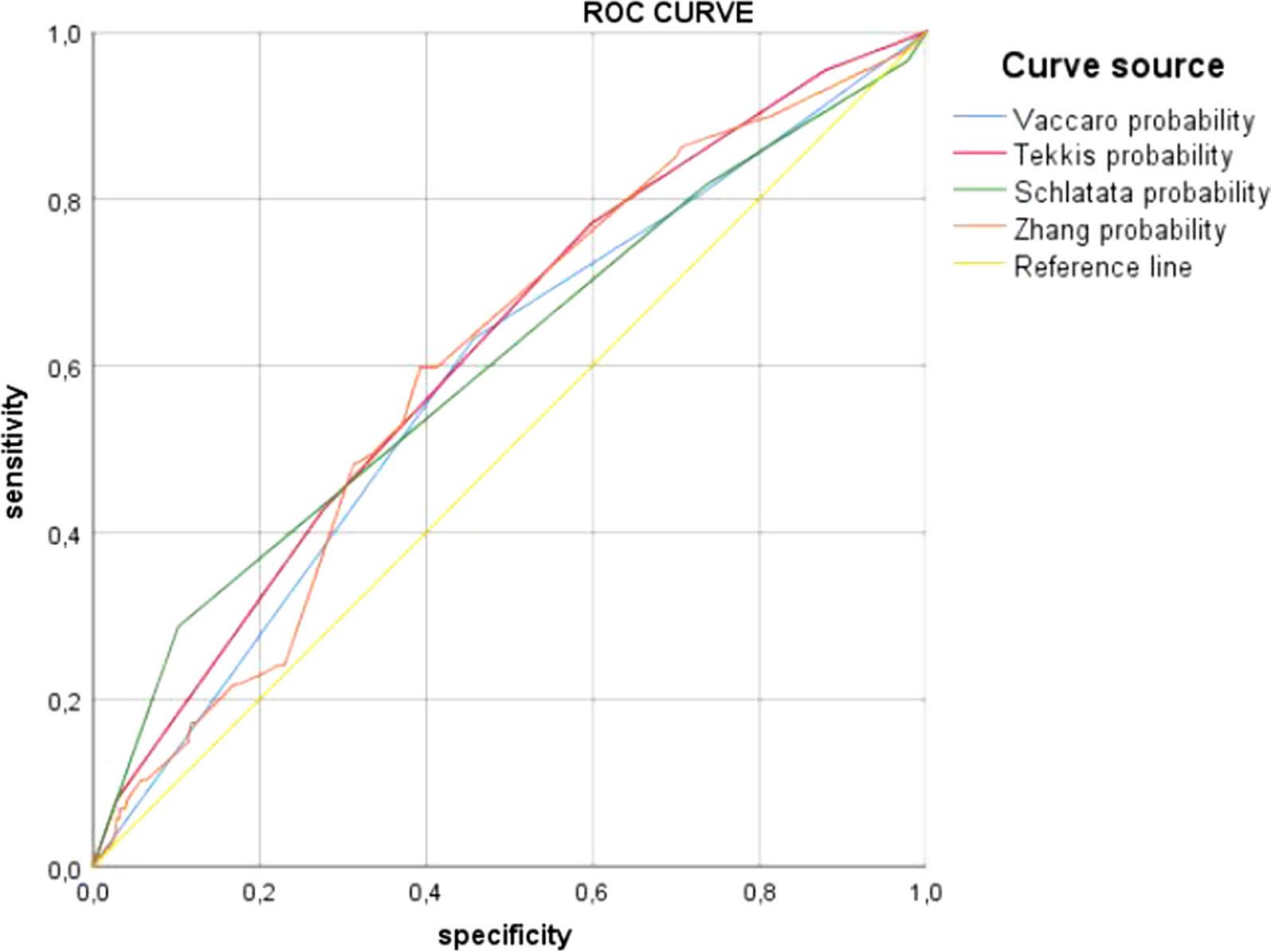
Comparison of area under the curve for all models

All the scores had an area under the curve (AUC) below 0,8. The Tekkis score had the highest AUC (0.617 95% CI [0.552; 0.683]). The AUC Schlachta’s score (0.602 CI [0.531; 0.673]) and Zhang’s score (0602 95% CI [0.537; 0.667]) were the same, while Vaccaro score had the lowest AUC of 0.585 95% IC [0.517; 0.652] (Fig. 1).

**Fig. 2:**
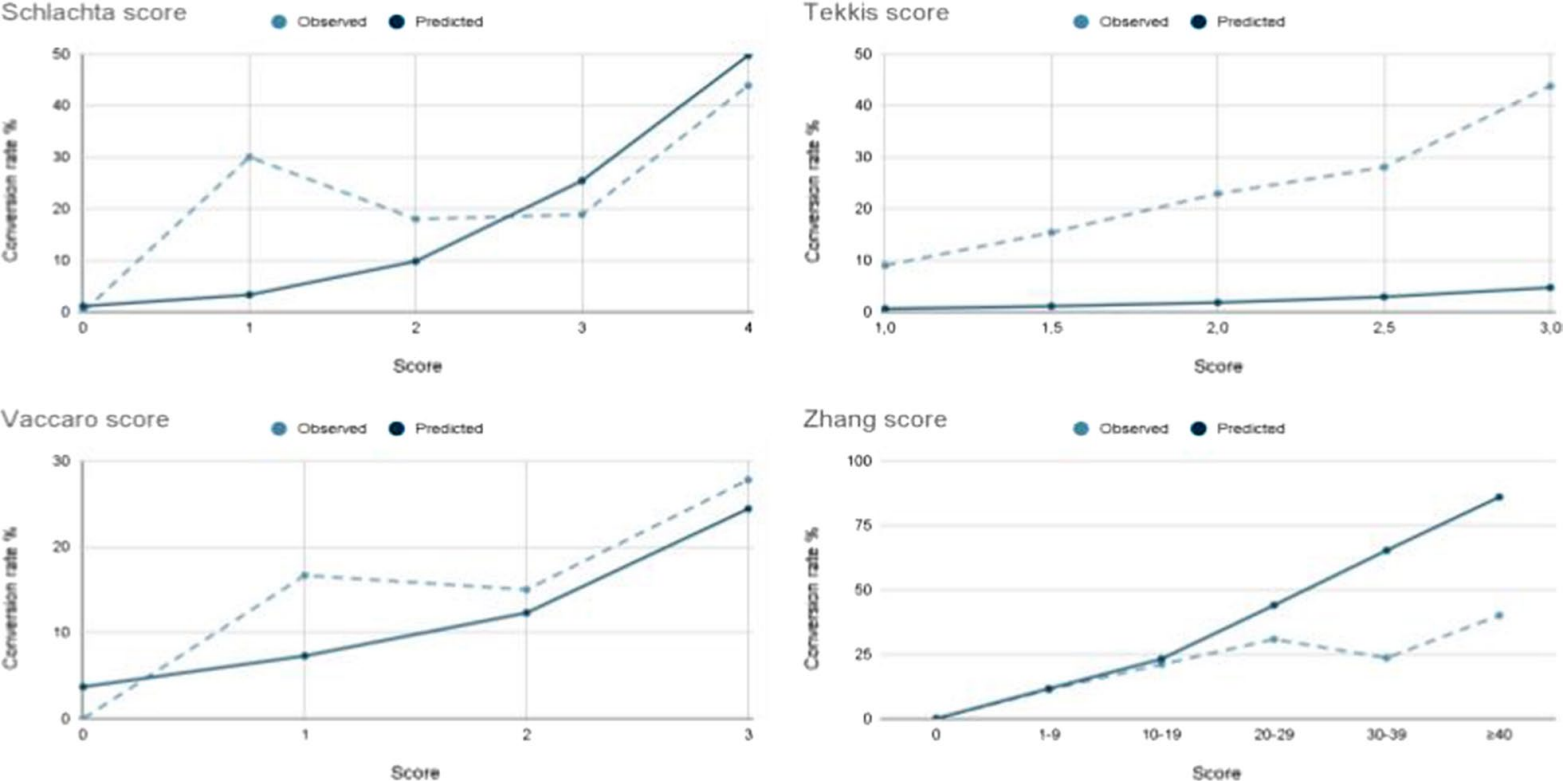
Observed versus predicted conversion rates for the four conversion scoring systems

Figure 2 shows the comparison between observed and predicted conversion rates for each score. Observed conversion rate was significantly higher than the conversion rates predicted using the Tekkis and Vaccaro models (X^2^ = 796,04; p = 0.0001 / X^2^ = 6,39; p = 0.01 respectively), and significantly lower than the conversion rate predicted by Zhang model (X^2^ = 14,44; p = 0.0001). There was no significant statistical difference between observed and predicted conversion rates for Schlachta’s model (X^2^ = 1,93; p = 0.16).

### Risk factors for conversion

Risk factors analysis included all the variables used by the 4 models studied.

In univariate analysis, the conversion rate was higher in patients with a BMI greater than 28 and a body surface area greater than 1.8 (36% vs. 16.7; p < 0.001 and 28.2 vs 14; p = 0.001 respectively). Seniors’ conversion rates were lower compared to younger surgeons (19.1% vs 33.3%; p = 0.006). Also, surgeons with less than 25 cases had significantly higher conversion rates (24.6% vs. 11.1% p 0.006). The T4 stage tumors were more related to conversion compared to other stages (47.6% vs. 20.2%. p = 0.003).

Age, sex, PS status, history of surgery, and neoadjuvant therapy were not related to the conversion rate. Disease-related factors such as tumor location and size, or metastasis presence, were not significantly related to conversion rate.

On multivariate analysis (Table 2), body mass index greater than 28, T4 tumors, and junior surgeons were significantly associated with a higher conversion rate.

**Table 2:** Univariate and multivariate analysis of predictive factors for conversion

Variable	Univariate analysis n (%)	p	Multivariate analysis OR [IC 95%]	p
Sex		0.361		
Male	61/264 (23.3)			
Female	26/136 (19.1)			
Age		0.05	1.59 (0.86—2.92)	0.14
< 60	18/117 (15.5)			
> 60	69/283 (24.5)			
PS status		0.91		
0–1	84/387 (21.8)			
2–3	3/13 (23.1)			
ASA score		0.423		
1–2	72/343 (21.0)			
3–4	13/50 (27.1)			
Tumor location		0.73		
Low	26/116 (22.4)			
Mid	24/124 (19.5)			
High	36/155 (23.4)			
Neoadjuvant CRT		0.28		
No	20/108 (18.7)			
Yes	67/283 (23.8)			
BMI		** < 0.001**	**2.48 (1.38—4.47)**	**0.002**
< 28	50/299 (16.7)			
> 28	**36/97 (36.0)**			
Body surface		**0.001**	1.79 (0.97—3.33)	0.06
< 1.8	24/173 (14)			
> 1.8	**62/221 (28.2)**			
Previous laparotomy		0.84		
No	83/380 (21.9)			
Yes	4/20 (21.1)			
Senior surgeon		**0.006**	**1.72 (0.94—3.14)**	**0.07**
No	**26/78 (33.3)**			
Yes	**61/322 (19.1)**			
Case number > 50		0.78		
No	80/365 (22)			
Yes	7/35 (20)			
Case number > 25		**0.009**	2.06 (0.94—4.52)	0.07
No	78/319 (24.6)			
Yes	**9/81 (11.1)**			
pT stage		**0.005**	**5.78 (2.10—15.90)**	**0.001**
0–3	**76/377 (20.2)**			
4	**10/22 (47.6)**			
M stage		0.401		
M0	78/347 (22.6)			
M1	9/52 (17.3)			
Tumor size		0.553		
< 6 cm	65/319 (20.5)			
> 6 cm	10/41 (24.4)			

Bold values indicate the variable with significant statistical significance

## Discussion

This study showed that the four predictive scores analyzed had low accuracy to predict conversion to open surgery. Even Though there was no significant difference between the predicted and observed conversion rates for the Schlachta score, the AUC was low (0.602) with a low performance to predict conversion for patients with lower scores (Fig. 2). None of the 4 models showed significant superiority over the others. We chose a bicentric design in two completely different settings to assess whether the predictive scores would be suitable for widespread use.

The conversion rate was 21.75%. Rates between 2 and 41% have been reported in the literature [15]. The main reported factors associated with conversion to open surgery in the literature are age, male gender, obesity, diverticulitis, history of abdominal surgery, and surgeon experience [15–17, 18]. A multivariate analysis of conversion risk factors showed a significant conversion increase in overweight patients and patients who were operated on for a locally advanced tumor or by junior surgeons. These risk factors are present in Schlachta et al., Tekkis et al., and Zhang et al. models [7–9, 13]. The seniority and experience of the surgeon were not described as risk factors by Vaccaro and al [6].

Although these variables are significantly common, the application of the models on our population resulted in a low ability to accurately predict conversion to open surgery. Similarly, Schlachta and Tekkis models also failed to predict conversion in another setting [8, 9, 13]. In 2010, Cima et al. tried to externally validate these 2 scores on a sample of 998 patients in one center. The areas under the curve of the 2 models were 0.62 [13], figures like those in our study.

Several hypotheses may explain why the scores failed to predict conversion in this study. First, Vaccaro et al., schlachta et al., and Tekkis et al. scores were validated in a heterogeneous population that also included patients who underwent colon resections and others diagnosis such as benign diseases (Diverticulosis, inflammatory bowel disease…). Therefore, their scores included variables that are not applicable specifically for rectal cancer surgery. Nevertheless, these scores were created to predict the risk of conversion for an individual patient based on specific characteristics, including tumor location and diagnosis. Therefore, these differences should not have an impact on the score accuracy. Interestingly, although these limitations do not apply to Zhang score that was created specifically for rectal cancer surgery, it also failed to predict conversion in this study with accuracy similar to the other scores. Second, the conversion is a result of several factors related to the disease, the surgeon, the surgical environment, and the patient himself [13]. Therefore, the risk factors considered in the scores may be not exhaustive. For example, previous studies have shown that pelvic dimensions had an impact on surgical difficulty and complications higher than gender or tumor stage [19, 20, 21, 22]. In addition, other important factors such as the surgeon's specialization in colorectal surgery or surgeon/center volume in colorectal surgery were related to conversion in previous studies [23]. Therefore, it is important to create scores that analyze as many relevant risk factors as possible. Finally, as stated by Cima et al., each institution has its own specific environment, infrastructure, organization, and system of care. Therefore, a predictive score developed in one institution may not be suitable for widespread use [13].

Limitations of the study include its relatively low sample compared to previous studies and the long period for patient inclusion. Another limitation was the differences in conversion to open surgery definitions between the studies and our study. We chose a widely accepted definition. However, this difference may have biased interpretation of the results.

## Conclusions

In conclusion, the four scores analyzed in this study had low accuracy in predicting the conversion to open surgery for laparoscopic rectal resection. Future research in the subject should analyze more specific variables, in a multicentric design to ensure generalizability of the results for daily surgical practice.

## Data Availability

Data and materials related to this study are available upon a reasonable request.

## Abbreviations

BMI: Body mass index
ASA: American Society of Anesthesiologists
STROBE: Strengthening the Reporting of Observational Studies in Epidemiology
CIL: Comity of Informatics and Liberty
TNM: Tumor-node-metastasis
ROC: Receiver operating characteristics
AUC: The area under the curve
CI: Confidence intervals

## References

[1] Greenblatt DY, Rajamanickam V, Pugely AJ, Heise CP, Foley EF, Kennedy GD (2011). Short-term outcomes after laparoscopic-assisted proctectomy for rectal cancer: results from the ACS NSQIP. J Am Coll Surg.

[2] Guillou PJ, Quirke P, Thorpe H, Walker J, Jayne DG, Smith AMH, Heath RM, Brown JM, MRC CLASICC trial group (2005). Short-term endpoints of conventional versus laparoscopic-assisted surgery in patients with colorectal cancer (MRC CLASICC trial): multicentre, randomised controlled trial. Lancet.

[3] van der Pas MH, Haglind E, Cuesta MA, Fürst A, Lacy AM, Hop WC, Bonjer HJ, COlorectal cancer Laparoscopic or Open Resection II (COLOR II) Study Group (2013). Laparoscopic versus open surgery for rectal cancer (COLOR II): short-term outcomes of a randomised, phase 3 trial. Lancet Oncol.

[4] Kayano H, Okuda J, Tanaka K, Kondo K, Tanigawa N (2011). Evaluation of the learning curve in laparoscopic low anterior resection for rectal cancer. Surg Endosc.

[5] Agha A, Fürst A, Iesalnieks I, Fichtner-Feigl S, Ghali N, Krenz D, Anthuber M, Jauch KW, Piso P, Schlitt HJ (2008). Conversion rate in 300 laparoscopic rectal resections and its influence on morbidity and oncological outcome. Int J Colorectal Dis.

[6] Vaccaro CA, Rossi GL, Quintana GO, Soriano ER, Vaccarezza H, Rubinstein F (2014). Laparoscopic colorectal resections: a simple predictor model and a stratification risk for conversion to open surgery. Dis Colon Rectum.

[7] Zhang G-D, Zhi X-T, Zhang J-L, Bu G-B, Ma G, Wang K-L (2015). Preoperative prediction of conversion from laparoscopic rectal resection to open surgery: a clinical study of conversion scoring of laparoscopic rectal resection to open surgery. Int J Colorectal Dis.

[8] Tekkis PP, Senagore AJ, Delaney CP (2005). Conversion rates in laparoscopic colorectal surgery: a predictive model with 1253 patients. Surg Endosc.

[9] Schlachta CM, Mamazza J, Seshadri PA, Cadeddu MO, Poulin EC (2000). Predicting conversion to open surgery in laparoscopic colorectal resections. A simple clinical model. Surg Endosc.

[10] Ebrahim S, Clarke M (2007). STROBE: new standards for reporting observational epidemiology, a chance to improve. Int J Epidemiol.

[11] Gérard J-P, André T, Bibeau F, Conroy T, Legoux J-L, Portier G, Bosset J-F, Cadiot G, Bouché O, Bedenne L, Française S, de Chirurgie Digestive (SFCD), Société Française d’Endoscopie Digestive (SFED), Société Française de Radiothérapie Oncologique (SFRO) (2017). Rectal cancer: French Intergroup clinical practice guidelines for diagnosis, treatments and follow-up (SNFGE, FFCD, GERCOR, UNICANCER, SFCD, SFED, SFRO). Dig Liver Dis.

[12] Fleshman J, Sargent DJ, Green E, Anvari M, Stryker SJ, Beart RW, Hellinger M, Flanagan R, Peters W, Nelson H, Clinical Outcomes of Surgical Therapy Study Group (2007). Laparoscopic colectomy for cancer is not inferior to open surgery based on 5-year data from the COST Study Group trial. Ann Surg.

[13] Cima RR, Hassan I, Poola VP, Larson DW, Dozois EJ, Larson DR, O’Byrne MM, Huebner M (2010). Failure of institutionally derived predictive models of conversion in laparoscopic colorectal surgery to predict conversion outcomes in an independent data set of 998 laparoscopic colorectal procedures. Ann Surg.

[14] Hosmer DW, Lemeshow S, Sturdivant RX (2013) Applied logistic regression. Wiley Series in Probability and Statistics.

[15] Schwandner O, Schiedeck TH, Bruch H (1999). The role of conversion in laparoscopic colorectal surgery: do predictive factors exist?. Surg Endosc.

[16] Crippa J, Grass F, Achilli P, Mathis KL, Kelley SR, Merchea A, Colibaseanu DT, Larson DW (2020). Risk factors for conversion in laparoscopic and robotic rectal cancer surgery. Br J Surg.

[17] Jayne D, Pigazzi A, Marshall H, Croft J, Corrigan N, Copeland J, Quirke P, West N, Rautio T, Thomassen N, Tilney H, Gudgeon M, Bianchi PP, Edlin R, Hulme C, Brown J (2017). Effect of robotic-assisted vs conventional laparoscopic surgery on risk of conversion to open laparotomy among patients undergoing resection for rectal cancer: the ROLARR randomized clinical trial. JAMA.

[18] Franko J, O’Connell BG, Mehall JR, Harper SG, Nejman JH, Zebley DM, Fassler SA (2006). The influence of prior abdominal operations on conversion and complication rates in laparoscopic colorectal surgery. JSLS.

[19] van der Pas MHGM, Deijen CL, Abis GSA, de Lange-de Klerk ESM, Haglind E, Fürst A, Lacy AM, Cuesta MA, Bonjer HJ, COLOR II study group (2017). Conversions in laparoscopic surgery for rectal cancer. Surg Endosc.

[20] Atasoy G, Arslan NC, Elibol FD, Sagol O, Obuz F, Sokmen S (2018). Magnetic resonance-based pelvimetry and tumor volumetry can predict surgical difficulty and oncologic outcome in locally advanced mid-low rectal cancer. Surg Today.

[21] Yamamoto T, Kawada K, Kiyasu Y, Itatani Y, Mizuno R, Hida K, Sakai Y (2020). Prediction of surgical difficulty in minimally invasive surgery for rectal cancer by use of MRI pelvimetry. BJS Open.

[22] Kim JY, Kim YW, Kim NK, Hur H, Lee K, Min BS, Cho HJ (2011). Pelvic anatomy as a factor in laparoscopic rectal surgery: a prospective study. Surg Laparosc Endosc Percutan Tech.

[23] Ackerman SJ, Daniel S, Baik R, Liu E, Mehendale S, Tackett S, Hellan M (2018). Comparison of complication and conversion rates between robotic-assisted and laparoscopic rectal resection for rectal cancer: which patients and providers could benefit most from robotic-assisted surgery?. J Med Econ.

